# Assessing the Levels of Awareness among European Citizens about the Direct and Indirect Impacts of Plastics on Human Health

**DOI:** 10.3390/ijerph18063116

**Published:** 2021-03-18

**Authors:** Jelena Barbir, Walter Leal Filho, Amanda Lange Salvia, Maren Theresa Christin Fendt, Rachel Babaganov, Maria Cristina Albertini, Alessandra Bonoli, Maximilian Lackner, Daniela Müller de Quevedo

**Affiliations:** 1 Faculty of Life Sciences, Hamburg University of Applied Sciences, 21033 Hamburg, Germany; walter.leal2@haw-hamburg.de (W.L.F.); maren.fendt@haw-hamburg.de (M.T.C.F.); Rachel.Babaganov@haw-hamburg.de (R.B.); 2 Graduate Program in Civil and Environmental Engineering, University of Passo Fundo, 99052-900 Passo Fundo, Brazil; 112737@upf.br; 3 Department of Biomolecular Sciences, University of Urbino Carlo Bo, 61029 Urbino, Italy; maria.albertini@uniurb.it; 4 Department of Civil, Chemical, Environmental and Materials Engineering, University of Bologna, 40131 Bologna, Italy; alessandra.bonoli@unibo.it; 5 Faculty of Industrial Engineering, University of Applied Sciences Technikum Wien, Hoechstaedtplatz 6, 1200 Vienna, Austria; maximilian.lackner@technikum-wien.at; 6 Clean Technology Center, Feevale University, 93525-070 Novo Hamburgo, Brazil; Danielamq@feevale.br

**Keywords:** plastics, human health, global assessment, awareness, microplastics, littering

## Abstract

There is an urgent need to assess European citizens’ perspective regarding their plastic consumption and to evaluate their awareness of the direct and indirect effect of plastics on human health in order to influence current behavior trends. In this study, the evaluation has been cross-related with scientific facts, with the final aim of detecting the most recommendable paths in increasing human awareness, reducing plastic consumption, and consequently impacting human health. A statistical analysis of quantitative data, gathered from 1000 European citizens via an online survey in the period from May to June 2020, showed that a general awareness about the direct impact of plastic consumption and contamination (waste) on human health is high in Europe. However, only a few participants (from a higher educational group) were aware of the indirect negative effects that oil extraction and industrial production of plastic can have on human health. This finding calls for improved availability of this information to general public. Despite the participants’ positive attitude toward active plastic reduction (61%), plastic consumption on a daily basis is still very high (86%). The most common current actions toward plastic reduction are plastic bag usage, reusage, or replacement with sustainable alternatives (e.g., textile bags) and selecting products with less plastic packaging. The participants showed important criticism toward the information available to the general public about plastics and health. This awareness is important since significant relation has been found between the available information and the participants’ decisions on the actions they might undertake to reduce plastic consumption. The study clearly showed the willingness of the participants to take action, but they also requested to be strongly supported with joint efforts from government, policies, and marketing, defining it as the most successful way toward implementing these changes.

## 1. Introduction

Humans are highly dependent on plastic materials since they are lightweight, cost-effective, durable, and efficient to produce. Therefore, plastics have replaced glass, wood, and metal in many products and nowadays have become a part of daily household supplies, technology, medical use, and packaging [1]. The use of plastics has dominated everyday life, especially post World War II. In 2019, global plastic production reached 359 million tons (Mt), of which 17% (62 Mt) was produced in Europe [2]. Of the total plastic production, two-thirds remains in the environment, air, soil, and water. Inevitably, fragments (microplastics) and leached-out components can enter and affect the human body [3,4]. Although use of plastic has many advantages, plastic leakage into the environment is nowadays an issue of increasing importance [5,6,7]. Major sinks for plastics are the oceans, where plastics partly swim on the surface and pollute beaches [8]. Marine currents collect floating plastics, e.g., in the Great Pacific garbage patch [9] and other spots the size of entire countries. Plastics can be found not only in the sea but also in polar regions [10]. Regarding Europe, the Mediterranean Sea is strongly affected [11]. On average, 135 (large) plastic items/km^2^ can be counted in the Western Mediterranean beaches [8]. The effect of plastics on the environment, particularly in the oceans, has been described as lethal to fish, birds, and mammals [12].

Besides direct landscape issues caused by plastic littering, plastic toxicity builds up on the individual to the global level, with many negative health consequences. Studies have found that plastics can interact with human cells, through particle and chemical toxicity and pathogen and parasite vectors [4].

Micro- and nanoplastic pollution in soil, marine, and freshwater ecosystems causes serious problems to living organisms, including humans [13,14,15,16,17]. Plastic particles have been found to damage the lung and the gut, crossing the blood–brain barrier or human placenta, leading to inflammation and damage to cells. Plastics leach toxic chemicals over the plastic lifecycle, which we are inevitably exposed to through inhalation and ingestion.

Besides what is mentioned above, plastics can release a variety of toxic compounds. Bisphenol A (BPA) is considered to be an endocrine disruptor with hormone-like properties, which can leach from plastic containers, and has been associated with health issues, inducing decreased male fertility, obesity, and increased puberty [18]. BPA is a precursor for polycarbonates and epoxy resins. There are also links of exposure to plastics and cancers, including breast and prostate cancer [1,18]. The toxins are so prevalent in our surroundings that one study found that 95% of participants in the United States (USA) had BPA in their urine [1]. To protect vulnerable populations such as babies and children from these health effects, the USA has banned the use of BPA in infant bottles and cups for toddlers [1].

However, many of these facts are only known to scientists, not reaching the general public. In many countries, people are unaware of the effects that these toxins play on their health and, therefore, are blindly exposed to and experience negative and harmful effects of the toxins mentioned. There is a need for environmentally minded behavior from ordinary citizens to reduce the release of plastics into the sea [19]. For examples, awareness among people [20] and public information campaigns [21,22,23] promote co-responsibility for plastics littering. Xu et al. [24] found that health consciousness and environmental awareness among consumers increases the purchasing of “green” products.

Considering the above facts, this study aims to understand the global awareness of European survey participants regarding the impacts of plastics on human health. The question “What is the level of European awareness regarding the negative impact of plastics on human health?” was formulated as a basis of this research. To answer this question, three specific objectives were developed: (a) to evaluate the awareness of people about existing direct and indirect impacts of plastics on human health; (b) to evaluate if gender, age, or education level influence their awareness and opinion; and (c) to recommend actions that could increase the awareness of people about the health threats posed by plastics.

To meet these objectives, the paper is structured as follows: Section 2 provides a short literature review of plastic production and its negative effect on human health and the environment. Section 3 is an overview of the methodology used, while Section 4 provides the results and discussion. Finally, Section 5 overviews the main conclusions of this research.

## 2. Theoretical Background

### 2.1. Plastic Production and the Environment

In recent years, global plastic production has increased significantly from two million tons in 1950 to around 360 million tons in 2018 (Eurostat, 2019). Their favorable physical-chemical characteristics make them the perfect material for a variety of industries, from food packaging, automotive, electronics, textile, and building to construction and medicine. At this rate of consumption, it is estimated that by 2050 up to 33 billion tons of plastic will accumulate globally [25].

Ten percent of the global waste generated is made up of plastics [26], and this prevailing and ubiquitous presence can consequently increase the risk of a large and uncontrollable release into the environment.

In Europe alone, 61.8 Mt of plastics was produced in 2018, while 29.1 Mt of post-consumer plastic waste material was collected, exposing that recycling is still very partial [2]. Plastic waste is not perfectly managed, with some escaping proper treatment, in part due to the large amount of single-use plastics that must be disposed of by individual users, and in part due to incorrect waste management systems.

Microplastics are also widespread. One can distinguish between secondary microplastics, which form in the environment from larger plastic waste by, e.g., attrition, and primary microplastics, which are released directly. An example of the latter are particles released from washing clothes, or microbeads. Such small plastic particles have many functions and different fields of application, because of their high-performance properties in emulsions, binding and forming films, or in abrasion processes. Microbeads are used as raw materials in many applications, for example, in textile, agriculture, or pharma industries, while in Europe, the cosmetic sector turns out to be the one with the highest demand [27]. Microbeads have now partly been replaced by other materials due to pressure from environmental protection groups. Another significant source of microplastics is tire attrition. Fibers deriving from the decomposition of textiles represent one of the most common pollutant microplastics. It is estimated that by washing six kilograms of fabric, up to 700,000 fibers are released, reaching enormous values of pollutant particles in water [28]. As mentioned above, plastic-based materials are pervasive and negatively impact both the ecosystem and human health. There is a direct relationship between the proliferation of plastics and their impacts on most ecological subjects and ecosystem services, locally and globally. This impact constitutes clear costs for the economy and human well-being [29], particularly in regard to the marine ecosystem.

It is estimated that up to 10 percent of plastic production is released, accumulating nearly 10 million tons a year on the surface or bed of seas and oceans. With the colonization of floating plastics by microorganisms, algae, and plants, the density of the plastic parts increases and they sink to the bottom of the ocean [30]. Macroplastic contamination usually affects large animals because of ingestion or entanglement within plastic debris, but aquatic organisms may be mainly contaminated by micro- and nanoplastics. For example, the majority of ingested particles in fish specimens were represented by fibers (70%) and hard plastics (20.8%) [31]. Moreover, fish may be contaminated after fishing during their storage and transportation in plastic packaging and containers. Plastic contamination may endanger human health, mainly in the case of its entrance in the food chain [32]: plastic particles have been in fact detected in various organisms from the bottom of the food chain, such as zooplanktons, to the highest levels, and humans definitely consume organisms and aliments that contain microscopic plastic debris [33].

### 2.2. Raising Human Awareness about Plastic Contamination and Its Effects on the Environment and Health

There is no doubt that a strong collective awareness of environmental issues has grown in recent times. Today, the ecological emergency is no longer perceived as a priority only for the élite, but has become a problem experienced by most people who take charge of it through acquisition of qualified knowledge. Recently, the willingness to learn specific environmental notions has grown substantially, including the belief that each individual has a strong impact on the environment. More and more people are adopting a careful and respectful lifestyle.

As mentioned previously, plastics are central to the economy due to their low cost and various functional properties. This universal material can cause serious danger to the environment and the health of consumers in different direct and indirect modes, and therefore growing worry about the environment and the impacts on human health has forced the industry to seek alternative materials [34]. The impact that plastic can have on human health has been the subject of studies by numerous researchers considering the toxicity of its components. The In Vitro Toxicity and Chemical Composition of Plastic Consumer Products study was conducted by researchers, who analyzed 34 articles composed of eight main types of polymers (polypropylene (PP), low-density polyethylene (LDPE), polyvinyl chloride (PVC), polyethylene terephthalate (PET), polyurethanes (PU), polystyrene (PS), and polylactide (PLA)), also intended for food. They were of commonly used objects such as plastic bottles, yogurt pots, and synthetic sponges. For each of them, some toxicity values were taken into consideration [35]. Specifically, the authors identified the effects of general toxicity in 6 out of 10 objects, oxidative stress in 4 out of 10 objects, and endocrine interference in 3 out of 10 products. The authors highlighted, in particular, that PVC and PU carried the highest degree of toxicity. By contrast, articles composed of PET and high-density polyethylene (HDPE) caused a low degree of toxicity.

The way in which plastic is currently produced, used, and disposed of does not allow one to reap the economic advantages of a more “circular” approach, and damages the environment. Mankind as a whole must urgently address the environmental problems that loom over the production, use, and consumption of plastic. For example, the millions of tons of plastic waste that end up in the oceans each year are one of the most obvious and alarming signs, resulting in growing concern in public opinion.

In September 2018, the European Union (EU) approved a plastic strategy that aims to change the design, construction, use, and recycling of plastic products. The purpose of this strategy is not to implement a “war” on plastic, but to foster a circular economy of plastic in which to treat this material in a sustainable and responsible way, so as to be able to stop the harmful effects and preserve the value of the chain of production [36]. The dual objective is therefore to protect the environment while simultaneously laying the foundations for a new economy of plastic materials, one in which the design and production fully respect the needs of reuse and recycling and products are developed with more sustainable and ecological materials [37].

## 3. Methodology

To reach the proposed aim of this study, a questionnaire of 20 questions was developed. It was divided into three sections: (1) for collecting background details (country, gender, age group, level of education, main plastic items consumed, and attitude toward this consumption); (2) for investigating the participants´ awareness regarding impacts of plastics on human health; and (3) for evaluating the efforts of the participants to reduce use of plastic and their willingness to change current practices and habits.

The approach used to assess the awareness of the impacts of plastics on human health was based on the report “Plastic & Health: The Hidden Costs of a Plastic Planet” [3], which presents the impacts according to the stages of the plastic lifecycle: extraction and transportation, refining and manufacturing, and consumer use and waste management. The source was also indicated in the survey for the respondents to look for more details, if desired, since the idea behind the questionnaire was to collect data for the study, while also providing information to respondents.

Options with the 5-point Likert scale were used to assess awareness on the topic. In addition to the questions focused on the stages of plastic lifecycle, a last question presented a list of diseases for respondents to associate with plastic. In addition to Azoulay et al. (2019), other sources were used for the list of health impacts [1,15,18,32].

The first list of questions was pre-tested with members of the Research and Transfer Centre “Sustainability and Climate Change Management,” and adjusted for conciseness and clarity. The survey was aimed to reach the general public, to cover all age groups, and to represent both genders equally. To reach different participant groups, the survey was distributed via different European faculty and scientific mailing lists related to sustainability (in order to reach participants with a high level of education). The survey was also shared with country members of the international H2020 project BIO-PLASTICS EUROPE. In addition, different private and professional social media channels were used, including Facebook, LinkedIn, and Instagram. The survey was made available for two months between May and June 2020. During this period, 1000 responses were collected from 25 European countries.

The data were analyzed using descriptive statistics, with frequency tables and measurements of central tendency and dispersion. To identify possible associations between the profile variables and the questions that refer to frequency, attitudes, the use of plastics, and knowledge and information regarding the impacts of plastic on human health, the chi-square test was used, considering the variables in the nominal scale (categorical). In the cases of a 2 × 2 table, the *p*-value of the Fisher’s exact test was consulted. To identify significant differences between the categories of the respondents’ profile in relation to the variables of information availability (impacts of extraction, transport, refining and manufacturing, consumption and use, and plastic waste), which were originally measured on an ordinal scale (Likert, 5 points), non-parametric tests were applied. The Mann–Whitney test was applied for comparisons between the categories of gender. The ordinal scale code is as follows: 5 = extremely aware; 4 = moderately aware; 3 = slightly aware; 2 = somewhat aware; 1 = not at all aware. The non-parametric tests were chosen according to the recommendations of Urdan [38] and Hair [39], since the scales of the variables were coded in the ordinal scale. For comparisons between the categories of age and education, the Kruskal–Wallis test was applied, followed by Mann–Whitney with Bonferroni correction, for variables, where the Kruskal–Wallis test indicated significant differences between the groups.

The gender variable was measured considering three categories, namely male, female, and others. For terms of association with the study variables, the category “others” was suppressed due to the low number of cases, which compromises the chi-square test, which has the restriction that at each crossing table the minimum frequency must be greater than 5, if the number of degrees of freedom is 2 or more. The statistical inference was performed considering a significance level of 5%. The software used for the analyses was SPSS V. 24.0.

## 4. Results and Discussion

### 4.1. Demographic Information

**Figure 1:**
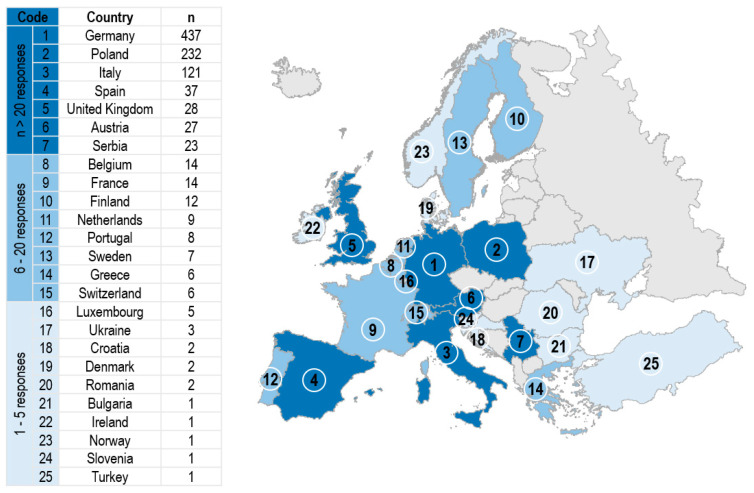
Location of all participating countries and number of participants per country.

Figure 1 presents 25 participating countries, as well as the number of responses received.

With regard to demographic characteristics, the sample is divided into 56.8% (*n* = 560) females, 43.4% (*n* = 430) males, and 0.6% (*n* = 6) who reported a different sex. This gender distribution was considered desirable for further comparison in behavior.

More than half of the sample was represented by respondents with a high education level, 30% (*n* = 301) having a master´s degree and 27% (*n* = 267) a PhD. One-third of the sample reported a middle education level with a bachelor’s degree (25%, *n* = 252) or a degree from a trade school (3%, *n* = 27). A low education level, “high school level or less,” was indicated by 15% of the sample (*n* = 153). To facilitate the analysis, the original five education levels were grouped in three: low education level (15.1%), middle education level (28.1%), and high education level (56.8%). In terms of age, the most represented groups were between 18–25 and 26–35 years (35.7% and 29.9%). The sample of this study was predominately comprised of young people having a higher education level, which might bias the final results. There is a significant relation between education and age groups (chi2, *p* < 0.001). Therefore, the analysis will be focused only on the education level and gender.

### 4.2. Assessing Frequencies and Modalities of Plastic Consumption in Europe

**Figure 2:**
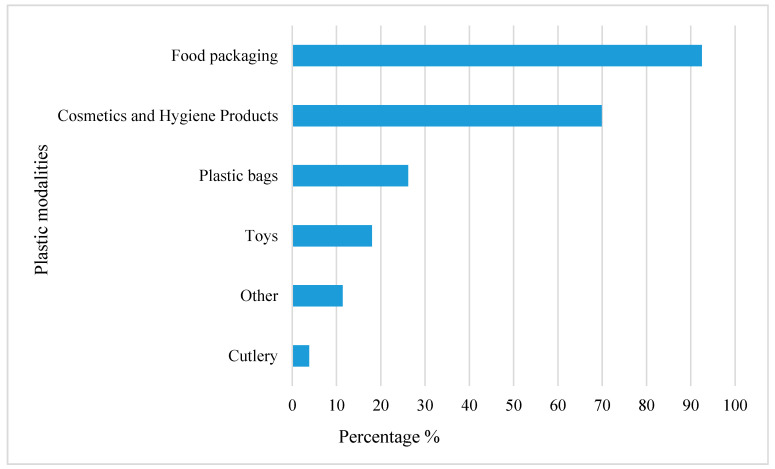
Assessment of plastic-based modalities used by European citizens derived from survey responses.

The main issue with plastics in the environment is not the materials themselves, but the management or rather the mismanagement of these plastics items, particularly low-value packaging, which leads to an accumulation of plastics in the environment. Considering that European citizens use plastic widely, the participants of the survey were asked to estimate which modalities of plastics they use and how often. The results (multiple answers were possible) showed that the two most common modalities of plastics among the participants are food packaging (*n* = 920, 92.5%) and cosmetics and hygiene products (*n* = 695, 69.9%). Those were followed by plastic bags (*n* = 260, 26.2%), toys (*n* = 177, 18%), and cutlery (*n* = 38, 3.8%), as shown in Figure 2. Food packaging was marked as a modality used by almost all participants. The importance of properly informing the consumers about the type of plastics used for food packaging and the effect it might have on their health should be considered urgent private and governmental priority, considering that chemicals from those materials migrate into the food that we consume daily [40].

The results exhibit that the usage of cosmetics and hygiene products is significantly related with gender (chi2, *p* < 0.001). There is a tendency to choose “yes” for both genders, but the results showed that the women chose this modality more frequently than men, underlining the fact that, in general, women are known to be more in contact with cosmetic packaging [41].

An interesting fact is that only 26.2% of all participants marked plastic bags as a plastic modality. In the last 10 years, a lot of social work and campaigns have been organized to reduce the usage of plastic bags. This tendency of participants not to mark plastic bags as a plastic modality shows clearly that joint efforts from producers and buyers can make significant changes in human behavior [42].

A significant relation was found with gender, showing the tendency for the response “no” for both genders, but again the results showed that women (*n* = 429, 76.6%) say “no” to plastic bags more frequently than men (*n* = 305, 70.3%). In addition, the usage of plastic bags is the only modality that is related with the education level and age of the participants, showing that those with a high school or bachelor’s degree (*n* = 222, 79.6%) are the groups who say “no” to plastic bags most frequently (chi2, *p* = 0.028). This might be related to the recent change in educational programs in both schools and universities in the last years.

The relative importance of the different plastic-based modalities in Figure 2 does not fully match production figures. The answers are more to be considered “front-of-the-mind” plastic products, which the respondents were thinking of, rather than an investigation of real mass-based consumption values. The different applications also vary in a lifetime, e.g., a plastic bag is typically used for less than five minutes, whereas toys provide service and value typically for several years.

The use of plastics is widely spread and the results showed that 86.6% (*n* = 861) of the participants use plastic products on a “daily basis,” 11.9% (*n* = 118) “occasionally,” and only 1.5% (*n* = 15) of the participants stated that they use plastic products “rarely.” According to Heidbreder [43], people are well aware of the negative impacts of plastics, yet due to convenience and habit, they are still very much in use.

**Figure 3:**
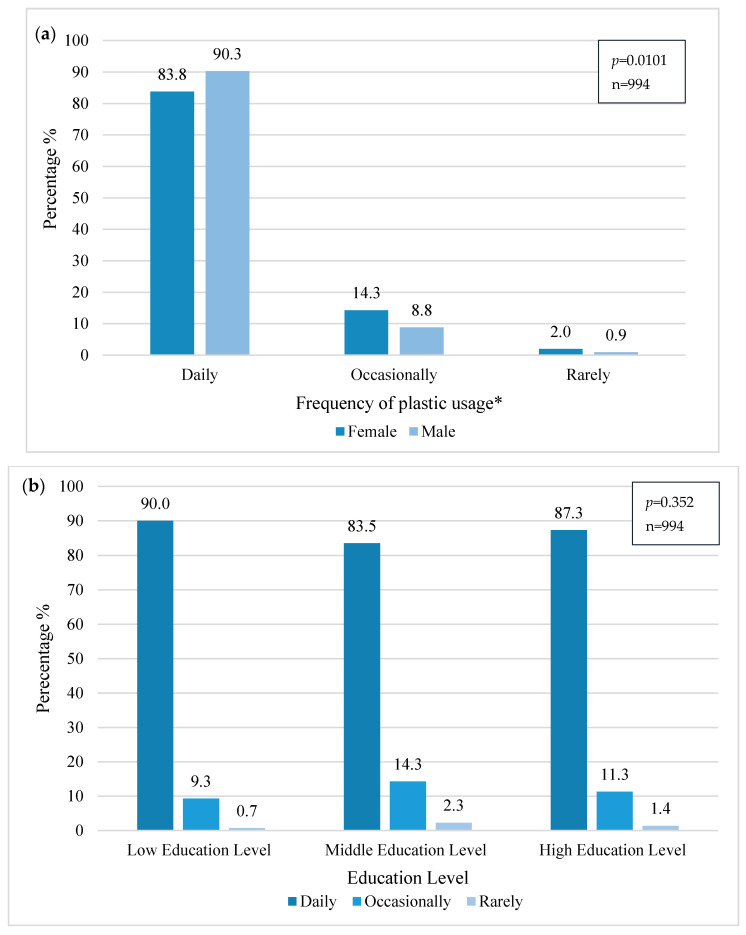
Frequency of plastic usage within the sample. (**a**) * Significant relation (chi2, *p* = 0.010) between gender and frequency of plastic usage. (**b**) No significant relation (chi2, *p* = 0.352) between education levels and frequency of plastic usage.

When related, a significant relation was found between gender and frequency of plastic usage (chi2, *p* = 0.010), showing that men (*n* = 392, 90.3%) use plastics more frequently than women (Figure 3). This can be discussed by considering the fact that in Europe, women are in direct contact with plastic products within the household [44], observing the problem that it produces, and therefore trying to avoid its daily usage.

Although no significant relation has been found between education groups and usage of plastics, there is a slight decrease in plastic usage in the middle education group, which also is found to most frequently reject the usage of plastic bags. 

### 4.3. Attitude toward Plastics Consumption

**Figure 4:**
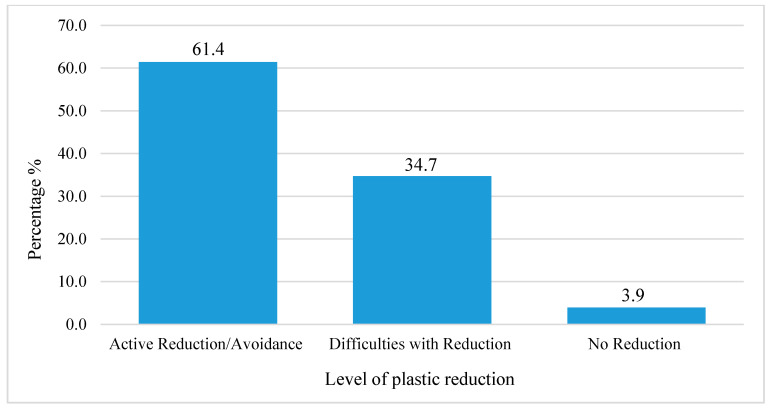
Attitude toward plastic consumption.

Although most participants are found to be in contact with plastics daily, their attitude toward plastic reduction is encouraging: 61.4% of all participants stated that they are actively reducing their usage of plastic on a daily basis (Figure 4), and 34.7% are aware of the problem and try to reduce plastic consumption, but they find it difficult since in many cases no alternatives are offered [9].

This attitude is encouraging, since only less than 4% do not show the attitude to implement changes in their behavior, while the other 96% are already modifying their behavior or trying to find alternatives. However, this result could be biased by the sample distribution, where over 50% are young and citizens having a higher education level. In any case, an overall positive attitude towards the reduction of plastic consumption among European citizens is the first step towards the successful implementation of behavioral changes [45]. Significant relationships have been found between both gender (chi2, *p* < 0.001) and education (chi2, *p* < 0.001) on the one hand, and attitudes of the participants on the other. 

The results showed that women (*n* = 311, 55.5%) are more eager to reduce plastic consumption than men (*n* = 188, 43.3%), who experience more difficulty in reducing plastic usage. Although education does not affect the frequency of plastic usage, it is significantly related with the attitude of the participants. Middle (*n* = 137, 49.1%) and high (*n* = 297, 52.6%) education levels were found to support reducing and actively avoiding plastics, while lower education levels (*n* = 65, 43.3%) experience more difficulties to reduce plastic usage or even state that there is no need for reducing plastic consumption.

Finally, when listing the actions to reduce plastic consumption (multiple answers were possible), almost all participants (*n* = 945, 95.1%) stated that they would bring their own bag to the supermarket (Table 1). This high awareness to reduce plastic bag usage was confirmed throughout the survey, in several questions, indicating that when marketing (e.g., TV, news, billboards), education (e.g., schools, universities), and governmental decisions (e.g., obligations to pay for a plastic bag) are combined and properly implemented, reduction in plastic consumption is possible.

Following replacing plastic bags with sustainable alternatives (reusable textile or paper bags), the participants expressed their willingness to buy products with less or no plastic packaging (*n* = 873, 87.8%). Around 70% of the participants also gave certain importance to the small plastic household accessories, including plastic cutlery, straws, or water purifiers. Finally, only 55.6% (*n* = 553) of the participants marked that they would shop in package-free stores. This can be explained due to the low availability and range of package-free stores, and the comparatively high price of the goods [46].

### 4.4. Availability of Information Regarding Plastics and Human Health

Recently, public attention and scientific research both move toward evaluating plastic consumption and its effect on human health. Therefore, the participants were asked about their opinion regarding the availability of this information to the general public and to define the most frequent sources of information.

**Figure 5:**
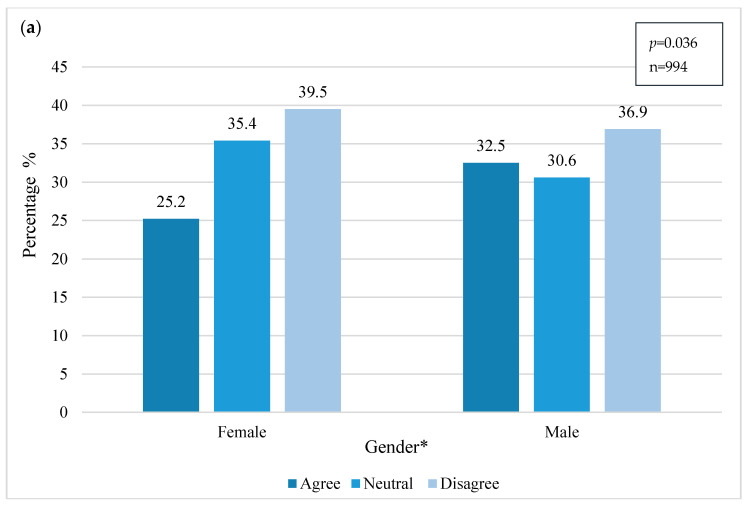

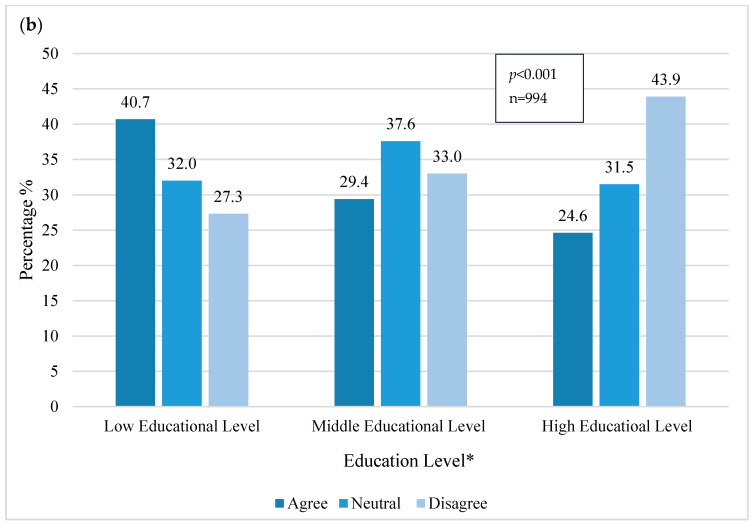
Availability of information regarding the impact of plastic on human health considering (**a**) gender and (**b**) education. * Significant relation (chi2, *p* = 0.036) between gender and opinion on available information. * Significant relation (chi2, *p* < 0.001) between education levels and opinion on available information.

Although many respondents had heard about the issue, they were still critical and required more information to be available. The results are related with gender (chi2, *p* = 0.036), showing that men are less critical and often agree that the currently available information on this issue is adequate and sufficient. However, over 75% of women remained neutral (*n* = 198) or disagreed (*n* = 221) with this fact (Figure 5).

In Figure 5, educational level is significantly related with the opinion and criticism of the sample regarding the availability of relevant information (chi2, *p* < 0.001). The Kruskal–Wallis test also indicates significant differences between the education groups (H (2): 24.378, *p* < 0.001). The results show that those with a lower education level are less critical and generally more likely to accept what is offered (*n* = 61). Those who hold a master’s and PhD generally rather disagree that the information offered regarding the negative impacts of plastics on the human health is satisfactory. These findings are aligned with studies conducted in different regions (*n* = 248) [7,47].

The study presented that most Europeans receive their information predominantly via news, followed by scientific articles, Google, and social media, and even through everyday communication with their families, friends, or colleagues (Table 2).

The fact that a high percentage of participants (48.8%) rely on scientific articles confirms why participants with higher education levels are more critical and aware of the problem. Many of the effects of the plastic lifecycle on human health are still under investigation, meaning that sometimes only laboratory results are available. The great proportion of the relevant information about this issue has been highly investigated; therefore, transfer to general public is still rather limited [48]. 

**Figure 6:**
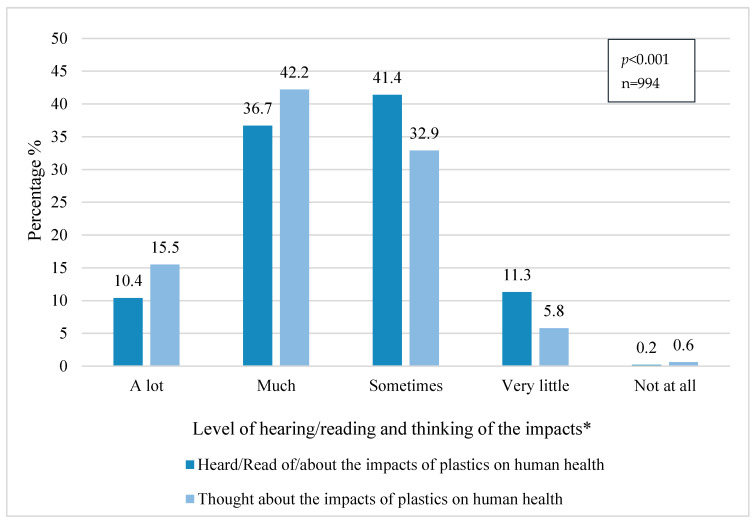
Receiving information and thinking about the impact of plastics on health. * Significant relation (chi2, *p* < 0.001) between receiving information and thinking about the impact of plastics on health.

Finally, a significant relation (chi2, *p* < 0.001) was found between how informed a person is and how much they reflect on the topic. Those who are informed “very little/a lot” about the impacts of plastics on human health also think “very little/a lot” about it. Figure 6 shows that the proportions for each category are similar in both variables.

Within the sample, 50% of the respondents heard and thought “a lot” or “much” about this topic, demonstrating that the awareness of people and information flow about this topic is relatively good. However, the results also clarify that it is highly recommended to provide clear facts and figures to the general public, since it will directly affect their behavior.

A strong relation between gender and “thinking about the impact of plastics on health” was found (chi2, *p* = 0.013), showing a slight tendency that women think more about this issue than men. A significant relation between educational groups and “thinking about the impact of plastics on health” was detected (chi2, *p* = 0.019), showing again that higher education groups think more often (a lot/much) about this issue (*n* = 359), which can be related to being better informed and closer to scientific sources of information [49,50]. 

### 4.5. Awareness about Direct and Indirect Impacts of Plastic Lifecycle on Human Health

Humans are exposed directly or indirectly to toxic chemicals and microplastics along the complete plastic lifecycle [46]. However, available information for general public has been mainly focused on direct human–plastic contact, food–plastic contact, and plastic waste in the environment [51]. Nevertheless, the impact of plastic on health begins at the oil wellheads, as plastic is a by-product of oil production [3].

The participants were asked to indicate how aware they are of the impact of the extraction and transportation of plastic on human health, and the results showed that only 36.7% of the participants were completely aware that this process can affect human health, while 22% had never heard about this (Table 3). These results are significantly related again with the education level (chi2, *p* = 0.027), where higher education groups are more aware about the negative impacts of the extraction of plastics on human health. The main information that reached the participants who are aware of it is that inhalation of toxins released by fracking is harmful for humans. Fewer participants were aware of the noise effect, and that communities that reside in closer proximity to fracking well sites are statistically more hospitalized than those residing further away [3].

Although research is still needed on various aspects of the human health impacts of the plastic production process (refining and manufacturing), many of the chemicals released into the environment coming into close contact with humans have impacts that are already known to be harmful. The participants showed higher awareness of the effect of this process on health, and only 11% of the participants were not aware of it (Table 3). Mainly the participants stated that they are aware that toxic chemicals cause eye and throat irritation, and headaches. In addition, they also related long-term exposures to chemicals with reproductive problems and cancers.

As predicted, participants were mainly aware of direct plastic consumption and plastic waste disposal effects on human health (Table 3), as well as direct contact with macro- and microplastics, probably due to the fact that available information about it is increasing everyday via channels that general public uses the most, such as the internet, news, and marketing [52]. When related with gender and education level, no significant relations were found on the overall topic, supporting the fact that this specific information is widely spread and available to general public. Despite constant investigations around the facts about the impacts of plastic production on health, and therefore available more to those with higher education levels, information on the negative impacts of plastic consumption on human health is readily available and consequently has a direct effect on human behavior. In the case of packaging and plastics in general, biodegradable and bio-based polymers (bioplastics) can be part of the solution [53].

Besides the direct negative effect of consuming microplastics (due to their intrusion and accumulation in human tissues), the microplastic particles in the environment were shown to accumulate toxins, and upon their ingestion through, e.g., seafood, humans are also exposed to these toxins [54]. This represents an important indirect negative effect on human health. Finally, over 90% of the participants are aware that plastic as waste can indirectly affect their health, and most of the participants were mainly concerned about the toxins from waste incinerations. With regard to specific questions, however, the Kruskal–Wallis test indicated significant differences between the education levels (H(2) = 15.609, *p* < 0.001).

Toxic chemicals produced during plastic production or plastic waste incineration can enter the human body through inhalation, while microplastics get in contact with the human body via ingestion or directly through skin contact. Therefore, the awareness of the participants regarding the consequences that the plastic lifecycle has on specific diseases or health problems was assessed (Table 4). The participants were asked to relate some of the health problems with plastics (directly or indirectly). The results displayed in Table 4 show that European participants strongly connect plastic and its lifecycle with cancer, followed by respiratory and reproductive problems, and cardiovascular and autoimmune diseases.

### 4.6. Recommendations Developed to Influence European Behavior towards Plastic Consumption to Protect Human Health

To develop some recommendations that could be implemented at the European level, the participants were asked to answer what should be done to increase human awareness about the effects that plastics may have on human health. In response, 86.7% of the participants stated that “promoting education about this topic in schools and universities” could greatly help future generations to be better informed and aware of the potential danger, 83.7% of the participants said that “proper labeling of the plastic packaging” would be helpful for instant decisions on product selection, and 53% of the participants stated that availability of information via media, and in general, should be increased.

Finally, the participants were asked who has the main responsibility for current trends in buying behaviors, public awareness, and reduction of plastic use and waste (Table 5). Around 50% of the participants stated for all categories that the responsibility is divided between the government and the public. This is a good representation to portray the willingness of individuals to implement and adapt the necessary changes in behavior, but only when properly guided by the government. In addition, the participants stated that buying behavior is more in the hands of the individuals. The participants felt that rising public awareness about plastics and its negative impacts on health, and overall plastic reduction, is more the responsibility of the government than individuals [55] (Table 5).

It is therefore imperative that governments, researchers, companies, and health authorities work cooperatively to increase the sustainable production, use, and disposal of plastics in order to limit their harmful effects on human health. The relevant information should be readily available for consumers to increase the awareness of products which are used and consumed and how they can play an integral part in health and well-being [18]. Control and responsibility not only reside with manufacturers and policy makers but, to a strong degree, also with consumers, who have a choice in purchasing and littering—or not.

**Figure 7:**
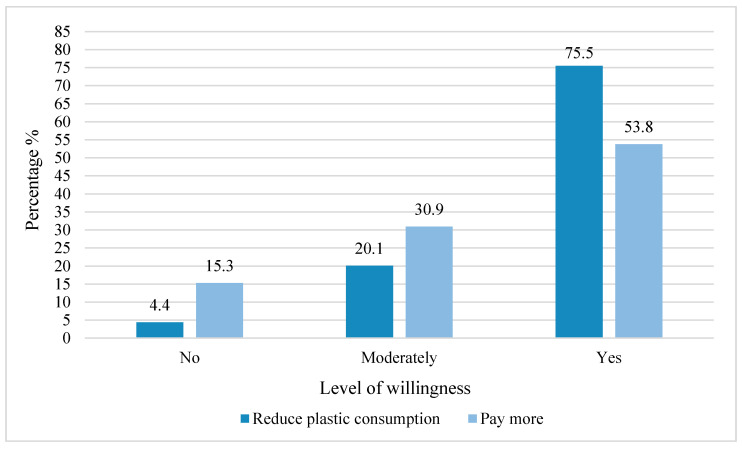
Willingness to reduce plastic consumption or to pay more for alternatives.

Many participants in this study expressed their willingness to reduce plastic consumption and pay more for the fossil-based plastics alternatives, e.g., bioplastics. The results showed that 75.5% of the participants expressed strong willingness to reduce plastic consumption even more in the future (Figure 7). This is an encouraging number, which points that European participants are on the right way toward behavioral change. However, these results must be interpreted with caution since the sample includes a higher rate of individuals with high educational level than the European population. Although this study provides an overall picture of the European situation, additional national European studies are needed to get detailed information from local people and consequently enable decision makers to provide the public with desirable solutions that match their attitudes and needs. In any case, a lot of work is still needed for successful transition from current plastic usage.

Again, a strong relation between gender and the willingness to reduce plastics has been found (chi2, *p* < 0.001), showing that women are more willing to reduce plastic consumption (*n* = 488) than men (*n* = 302).

## 5. Conclusions

This is the first study to investigate the perception of European participants regarding health risks from plastics. Findings from 1000 questionnaires indicate that there is basic knowledge regarding this issue (mainly in groups with higher education), but the problem is still underestimated among the general public. Although this study offers insight into the overall European situation, it is recommended that various local European studies be conducted to address the main issues of local people and offer them solutions that will match their needs. Better communication is desirable to increase citizens’ awareness of the presence of macro-, micro-, and nanoplastics in the environment, and the direct and indirect consequences it can cause to nature and humans. The study showed that gender has an important role when dealing with plastics, showing that women in general act more responsibly when consuming and disposing of plastics. Raising awareness among the general public is a crucial step in reducing plastic littering and pollution. Bioplastics could be a part of the solution to improve the plastic lifecycle; however, further investigation and research on additives and production of bio-based plastics are still required. Finally, a change in the attitude of consumers with regard to plastic consumption and littering, in combination with improved initiatives by the government, and the development of good policies are imperative to alleviate health problems from plastic waste in the environment.

## Figures and Tables

**Table 1:** Actions to reduce plastic consumption

Actions That Consumers Consider Taking to Reduce Plastic Consumption	%
Bringing their own bag to the supermarket	95.1
Buying products with less or no packing	87.8
Avoiding plastic cutlery when ordering takeaways or at catering events	74.4
Refusing plastic straws at restaurants and bars	73.1
Reducing the use of nondisposable plastic	72.9
Using water purifiers (avoiding plastic bottles)	69.4
Buying at package-free stores	55.6

**Table 2:** Source of information regarding plastic consumption and health.

Source of Information	%
News	73.0
Scholarly articles	48.8
Google	44.7
Friend/relative/colleague	44.9
Social media	42.9
Other	12.8
Never searched before	1.3

**Table 3:** Awareness about the direct and indirect impact of plastics on human health.

Question	Not Aware	Somewhat Aware	Aware
Impacts of plastic production (starting at the oil wellheads) on human health	22.1%	41.2%	36.7%
Impacts of plastic production (refining and manufacturing) on human health	11.0%	37.0%	52.0%
Impacts of consumer use of plastics on human health	6.7%	33.7%	59.6%
Impacts of plastic waste disposal and management on human health	6.9%	33.9%	59.2%

**Table 4:** Awareness about the impact of the plastic lifecycle on the development of specific diseases/health problems.

Health Impact in %	Aware	Somewhat Aware	Not Aware
Cancer	59.2	34.1	6.7
Respiratory problems	48.2	38.9	12.9
Reproductive problems	40.0	43.1	16.9
Chronic inflammation	32.1	49.3	18.6
Cardiovascular disease	28.9	48.3	22.8
Autoimmune disease	25.9	45.1	29.1
Mental health effects	22.5	44.9	32.6
Stroke	20.5	46.5	33.0
Inflammatory bowel disease	19.9	47.6	32.5
Rheumatoid arthritis	18.0	42.6	39.4
Diabetes	16.0	40.0	44.0

**Table 5:** Responsibility to reduce plastics: government vs. individual.

Responsibility in %	Government	Equal	Individual
Buying behavior	16.0 (14.6 F/17.7 M)	50.4 (78.6 F/72.8 M)	33.6 (6.8 F/9.4 M)
Awareness on plastics and health *	44.4 (40.4 F/49.5 M)	47.9 (58.8 F/48.4 M)	7.7 (0.9 F/2.1 M)
Reduce use and availability of plastics *	44.9 (42.0 F/48.6 M)	46.1 (56.3 F/47.5 M)	9.1 (1.8 F/2.7 M)

* Significant relation (*p* = 0.003) (chi2, *p* = 0.003) between gender (F: female; M: male) and responsibility about awareness and plastic reduction (chi2, *p* = 0.003), receiving information and thinking about the impact of plastics on health (chi2, *p* = 0.006).

## Data Availability

Data available on request due to restrictions e.g., privacy or ethical.
